# Sickle Cell Disease: Role of Oxidative Stress and Antioxidant Therapy

**DOI:** 10.3390/antiox10020296

**Published:** 2021-02-16

**Authors:** Rosa Vona, Nadia Maria Sposi, Lorenza Mattia, Lucrezia Gambardella, Elisabetta Straface, Donatella Pietraforte

**Affiliations:** 1 Biomarkers Unit, Center for Gender-Specific Medicine, Istituto Superiore di Sanità, Viale Regina Elena 299, 00161 Rome, Italy; rosa.vona@iss.it (R.V.); nadia.sposi@iss.it (N.M.S.); lucrezia.gambardella@iss.it (L.G.); 2 Department of Clinical and Molecular Medicine, “La Sapienza” University, 00161 Rome, Italy; lorenza.mattia@uniroma1.it; 3 Endocrine-Metabolic Unit, Sant’Andrea University Hospital, 00189 Rome, Italy; 4 Core Facilities, Istituto Superiore di Sanità, Viale Regina Elena 299, 00161 Rome, Italy; donatella.pietraforte@iss.it

**Keywords:** sickle cell disease, hemoglobin, oxidative stress, antioxidants, red blood cells

## Abstract

Sickle cell disease (SCD) is the most common hereditary disorder of hemoglobin (Hb), which affects approximately a million people worldwide. It is characterized by a single nucleotide substitution in the β-globin gene, leading to the production of abnormal sickle hemoglobin (HbS) with multi-system consequences. HbS polymerization is the primary event in SCD. Repeated polymerization and depolymerization of Hb causes oxidative stress that plays a key role in the pathophysiology of hemolysis, vessel occlusion and the following organ damage in sickle cell patients. For this reason, reactive oxidizing species and the (end)-products of their oxidative reactions have been proposed as markers of both tissue pro-oxidant status and disease severity. Although more studies are needed to clarify their role, antioxidant agents have been shown to be effective in reducing pathological consequences of the disease by preventing oxidative damage in SCD, i.e., by decreasing the oxidant formation or repairing the induced damage. An improved understanding of oxidative stress will lead to targeted antioxidant therapies that should prevent or delay the development of organ complications in this patient population.

## 1. Introduction

Sickle cell disease (SCD) is an inherited hemoglobinopathy and the most common severe monogenic disorder in the world. The United Nations (UN) and the World Health Organization (WHO) define the inherited blood disorders such as SCD as a global health problem, since there are more than 300,000 births annually affected [1,2,3].

This disease prevails in large areas of sub-Saharan Africa, the Middle East, India, the Caribbean, South and Central America, some countries along the Mediterranean Sea, as well as the United States and Europe. The global distribution of SCD is driven by two factors: (i) selection for carriers through their survival and (ii) population movements [4,5,6].

SCD is caused by a single nucleotide substitution (GTG for GAG) at the sixth codon of the β-globin gene, which is located on the short arm of chromosome 11 [7]. This induces the substitution of valine for glutamic acid at the sixth amino acid position in the β-globin chain, leading to the production of abnormal HbS (sickle hemoglobin), which has the propensity to polymerize under conditions of low oxygen saturation, such as occur in the microcirculation [8]. HbS polymerization is the primary event in SCD. Repeated polymerization cycles cause irreversible damage to red blood cell (RBC) deformability, while a single polymerization results in reversible decreased RBC deformability and increased mechanical fragility [9].

In addition to homozygous sickle cell disease (HbSS), other forms of such sickling hemoglobin anomalies exist, such as HbSC disease and HbSβ-thalassemia [10]. Repeated sickling of the RBCs results in membrane fragility and hemolysis, ischemia-reperfusion, occlusion of post-capillary venules and infarction [11].

Sickle cell disease is a chronic disease that has detrimental effects on the entire body and requires a multidisciplinary team for management.

In addition to HbS polymerization, vessel occlusion and hemolytic anemia, which play a central role in the pathophysiology of SCD, vascular-endothelial dysfunction, functional nitric oxide deficiency, inflammation, oxidative stress, reperfusion injury, hypercoagulability, increased neutrophil adhesiveness and platelet activation can lead to several complications [4].

A wide variability was observed in the clinical severity of SCD, as well as in the life expectancy [12]. Genetic variants controlling the expression of the HbF (fetal hemoglobin) genes and coinheritance of the α-thalassemia gene are associated, on average, with milder SCD phenotypes [12]. The role of other potential genetic modifiers is less clear.

The health and survival of children with sickle cell disease has been improved considerably by penicillin prophylaxis, pneumococcal immunization, advent of newborn screening and education about disease complications.

Wider use of transfusions, hydroxycarbamide and newer therapeutic approaches have offered hope for improved health-related quality of life and decreased mortality. Nevertheless, even with the best of care, life expectancy of affected adults is still reduced by about 30 years.

Pathological events occurring in sickle cell disease increase the free-radicals generation through activation of pro-oxidant enzymes, release of free hemoglobin, and heme induced by hemolysis which foster Fenton reaction, modification of mitochondrial respiratory chain activity and RBC auto-oxidation [10,13,14]. Excess of free radicals contributes to increased oxidative stress in RBCs, endothelial cells (ECs), neutrophils and platelets, which manifests as multiorgan vasculopathy.

Oxidative stress is defined as the imbalance between the levels of reactive oxygen species (ROS), reactive nitrogen species (RNS) and antioxidants activity or concentration. ROS derive from the reduction of molecular oxygen and include radical species, such as the poorly reactive superoxide anion radical (O_2_^•−^) and the strong reactive hydroxyl radical (^•^OH), as well as non-radical oxidants such as hydrogen peroxide (H_2_O_2_), hypochlorous acid (HClO) and hypobromous acid (HOBr) [11,14,15]. Similarly, RNS include radical species, such as the poorly reactive nitric oxide (^•^NO) and nitrogen dioxide (^•^NO_2_) and non-radical species such as nitrous acid (HNO_2_), dinitrogen trioxide (N_2_O_4_) and peroxynitrite (ONOO^−^). The latter, formed by the interaction between ^•^NO and O_2_^•−^, can induce irreversible modification of the activity and function of several key intracellular targets through the formation of strong oxidizing radicals such as ^•^NO_2_ and carbonate radical (CO_3_^•^). Moreover, peroxynitrite can induce the oxidation and nitration of sensible key target compounds, such as thiols, damaging cell membranes and mitochondria, causing DNA strand breakage and apoptosis [16]. The oxidation of biological molecules such as proteins, lipids, carbohydrates and DNA persists when it is not neutralized by the defense mechanisms, leading to impaired intracellular signaling, cellular dysfunction and death [10,11].

To counteract ROS and RNS, both non-enzymatic and enzymatic defense mechanisms have evolved [10,11]. Non-enzymatic antioxidants include ascorbic acid, glutathione, tocopherols, carotenoids, riboflavin and microelements such as zinc [10]. Enzymatic antioxidants include superoxide dismutase (SOD), catalase (Cat), glutathione peroxidase (Gpx), glutathione reductase, glutaredoxin (Grx), thioredoxin/thioredoxin reductase system and peroxiredoxins (Prx) [10].

Due to the high levels of oxidative stress, levels of both enzymatic and non-enzymatic antioxidants are reduced in SCD [16,17,18]. A wide range of non-enzymatic antioxidants has been found to be deficient in red blood cells, mononuclear cells, and platelets of SCD patients. They include glutathione, vitamin E and C, β-carotene, and plasma retinol [16,17,18,19]. Serum and plasma levels of the enzymatic antioxidants SOD, Gpx and Cat are also diminished [16,17,18,19].

The emerging idea for new SCD therapeutic approaches is that vasculopathy, adhesion events and inflammation, formation of dense RBCs, as well as oxidative stress might constitute new pharmacological targets. Oxidative stress is an important feature of SCD. In fact, reactive oxidizing species and the (end)-products of their oxidative reactions are potential markers of disease severity, thus representing targets for antioxidant therapies.

This review summarizes our current understanding on the mechanisms of oxidative stress in SCD and discusses the involvement of reactive oxidizing species in the SCD pathophysiology and management.

## 2. Source of ROS in SCD

In SCD, reactive oxidizing species are generated by sickle RBCs as well as by activated leukocytes, platelets, ECs, and plasma enzymes. Several mechanisms contribute to ROS and RNS formation in tissues of SCD patients such as (i) increased activity of nicotinamide-adenine dinucleotide phosphate (NADPH) oxidase and endothelial xanthine oxidase (XO) [8,10], (ii) HbS autoxidation [11], (iii) heme and iron release, (iv) increased asymmetric dimethylarginine (ADMA) [12,13], and (v) uncoupling of nitric oxide synthase (NOS) activity and decreased ^•^NO bioavailability [20].

### 2.1. Increased Activity of Several Oxidases

It has been demonstrated that in SCD, the enzymes NADPH oxidase, XO and uncoupled endothelial nitric oxide synthase (eNOS) can generate ROS in the vascular compartment [21,22,23,24].

NADPH oxidase is the major O_2_^•−^ producing enzyme in leucocytes, vascular endothelial cells and RBCs. ROS produced by activated leucocyte NADPH oxidase contribute to the hemolysis associated with infections or vessel occlusive crises [23]. The O_2_^•−^, derived from endothelial cell NADPH oxidase, contributes to the pro-inflammatory and pro-thrombogenic responses associated with SCD [24]. In RBCs, NADPH oxidase activity is regulated intracellularly by protein kinase C and Rac GTPases and extracellularly by signaling factors such as transforming growth factor β1 and endothelin-1 present in the plasma from SCD patients [24]. ROS derived by RBC NADPH oxidase may cause direct oxidative damage to a variety of subcellular structures, reducing RBC deformability and resulting in increased RBC fragility and hemolysis [24].

XO represents a potent source of superoxide O_2_^•−^ and H_2_O_2_, and its activity is increased in the plasma of SCD patients. The source of XO is not completely clear, but episodes of hypoxia/reoxygenation in SCD patients can stimulate the release of this enzyme from the liver into the circulation. Increased circulating XO can then bind avidly to vessel luminal cells and impairing vascular function and creating an oxidative milieu [25].

### 2.2. HbS Autoxidation

Normal RBCs continuously generate ROS during oxygenation/deoxygenation cycles occurring in the circulation. The oxygen exchange physiologically generates a continuous slow autoxidation of oxygenated Hb (ferrous, Hb-FeII) producing O_2_^•−^ and methemoglobin (ferric, Hb-FeIII), which no longer binds oxygen, at a rate of 0.5–3% per day. The spontaneous and enzymatic O_2_^•−^ dismutation forms H_2_O_2_, but both these species are neutralized by the efficient RBC antioxidant system involving both non-enzymatic low molecular weight antioxidants (glutathione, ascorbic acid and vitamin E) and enzymatic antioxidants (SOD, Cat, GR, Prx2 and Gpx). These antioxidant activities, coupled to the methemoglobin reductase-dependent reduction of Hb-FeIII to Hb-FeII, preserve RBCs integrity and function. Under conditions of oxidative stress, ROS are produced in greater quantities in normal RBCs, which by activating the pseudo-peroxidase cycle detoxify the generated oxidants leading to the complete consumption of H_2_O_2_.

**Figure 1:**
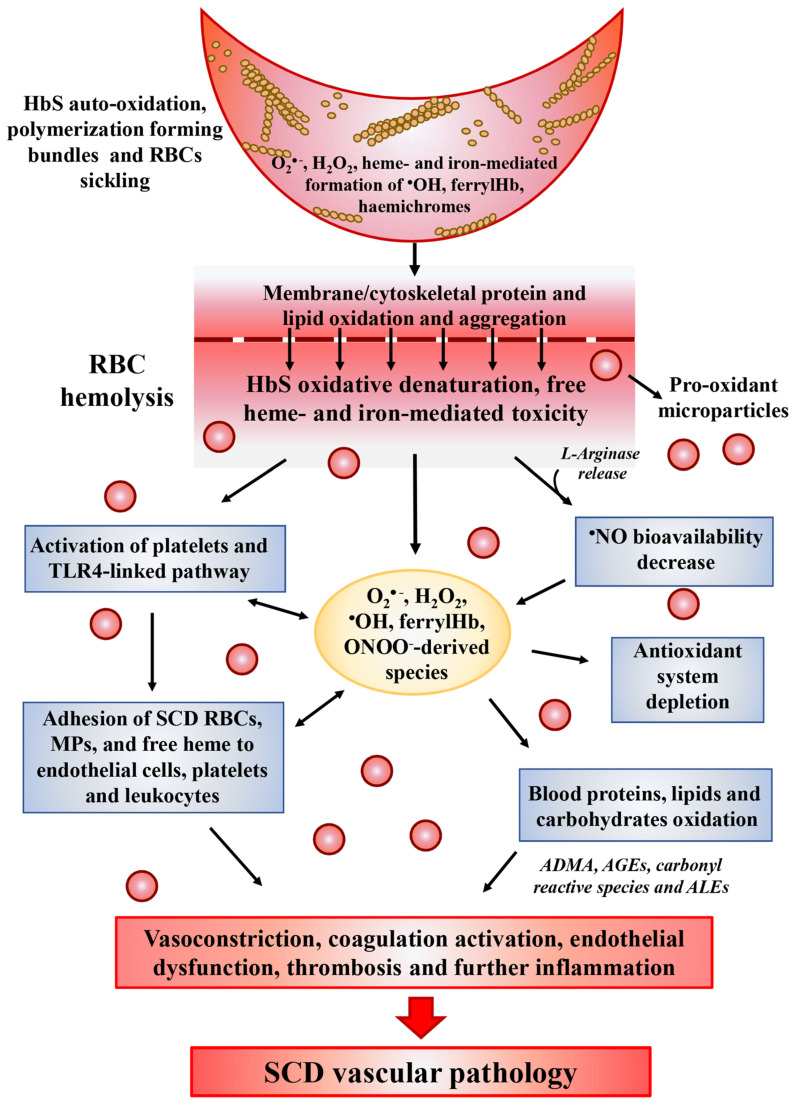
Pathophysiological effects of oxidative stress in sickle cell disease (SCD). Following oxygenation/deoxygenation cycles, HbS in RBCs undergoes autoxidation generating reactive oxidizing H_2_O_2_ and the release of free heme and iron. Heme and iron are key players in SCD oxidative damage by (i) catalyzing the generation of strong oxidizing species, such ^•^OH and ferrylHb through the H_2_O_2_-dependent Fenton reaction; (ii) boosting platelets activation and adhesion to endothelial cells; (iii) stimulating the Toll-like receptor-4 (TLR4) in endothelial cells, and promoting inflammasome activation and cytokines production (IL-1β, IL-6, IL-18, TNF-α) through NF-κB-linked pathways, (iv) activating neutrophils, leading to the release of neutrophil extracellular trap which can also affect endothelial cells and act as a scaffold for platelets and RBCs; (vi) favoring the expression of adhesion molecules (VCAM-1, ICAM-1, E-selectin, P-selectin) that are all markers of endothelial dysfunction and function as receptors for leukocyte (lymphocytes, neutrophils and monocytes); and (vi) stimulating the blood coagulation, inducing the exposure of intraluminal tissue factor in endothelial cell boosting the coagulation cascade through binding of tissue factor with Factor VIIa. Importantly, the vascular availability of ^•^NO, which has well known vasodilating, anti-thrombotic, and anti-inflammatory properties, is decreased by the hemolysis-mediated release of (i) HbS and free heme, which can bind ^•^NO in plasma, (ii) the enzyme L-arginase, which degrades the nitric oxide synthase substrate L-arginine to ornithine, and (iii) the O_2_^•−^ enzymatically produced by ROS-generating enzymes (NADPH oxidases, xanthine oxidase and uncoupled NOS) in activated leukocytes and platelets. In this latter case, the fast reaction between ^•^NO and O_2_^•−^ produces the strong oxidant peroxynitrite (ONOO^−^) and its derived oxidants (such as ^•^OH, ^•^CO_3_, ^•^NO_2_). The pro-oxidant status of SCD blood induces the depletion of both extra- and intra-cellular antioxidant defenses. The enhanced release of MPs by SCD RBCs could further exacerbate inflammation and oxidative stress, being overloaded with pro-oxidants molecules (methemoglobin, heme/iron and their derived oxidation products). All these combined pathways contribute to activate a vicious circle, which amplifies the formation of ROS from heme/iron and in cells activated as a result of inflammation. Moreover, it inhibits antioxidants depletion or in some cases boosts their replenishment (see the text) and promotes the oxidation of intra- and extracellular targets, i.e., proteins and lipids leading to the formation of asymmetric-dimethyl arginine (ADMA). In particular, new carbonyl groups can be introduced into proteins by: (i) direct oxidation of lysine, arginine, proline and threonine residues; (ii) reaction of oxylipin carbonyls bearing α,β-unsaturated bonds, such as a number of aldehydes (e.g., acrolein, malondialdehyde [MDA], 4-hydroxy-2-nonenal [HNE], 4-hydroxy-2-hexenal (HHE)) and some isoprostanes deriving from the peroxidation of polyunsaturated fatty acids (PUFAs), with lysyl residues; (iii) reaction of reducing sugars and their oxidation products (e.g., ketoaldehydes like methylglyoxal, deoxyosones like 3-deoxyglucosone and ketoamines) with lysyl residues [37]. Compounds in group (ii) are referred to as “advanced lipoxidation end-products” (ALEs). Compounds in group (iii) are referred to as “advanced glycoxidation end-products” (AGEs). This inclusive definition should be preferred over the widespread definition of “advanced glycation end-products”. Oxylipin carbonyls bearing α,β-unsaturated bonds, such as acrolein, MDA, HNE, HHE and some F2-isoprostanes deriving from the endocyclization of lipid hydroperoxyl radicals are collectively referred to as “reactive carbonyl compounds” [38]. Reactive compounds generated by these mechanisms participate in the vicious cycle of leukocyte and endothelial cell activation, leukocyte aggregation with RBCs/MPs and platelets, and eventually vascular occlusion.

In SCD, intravascular hemolysis results in the toxic accumulation of free HbS and heme in the plasma (Figure 1). Compared to normal Hb, HbS molecules are highly unstable in particular under hypoxic condition and more prone to autoxidation [26,27]. The rate of HbS autoxidation has been calculated to be about 2 times faster than that of normal Hb, resulting in the increase of about 2 times the generation of O_2_^•−^, H_2_O_2_, ^•^OH and lipid oxidation products [28,29]. This exacerbated pseudo-peroxidase cycle is followed by heme release and iron loss, both able to amplify oxidative reactions. In addition, the autoreduction of ferryl back to ferric heme is slower than that of normal Hb, leading to a longer lived and more damaging free ferryl Hb and to free ferryl radical. The latter has been shown to migrate and induce further damage in the protein, including the irreversible oxidation and dimerization of Cysβ93, as well as to induce, in target cells, damage and dysfunction in other biological organelles, such as in the mitochondria likely, contributing to SCD-induced vascular pathology [29]. In SCD, as well as in other hemolityc disorders, the high amounts of plasma hemoglobin is in the ferrous form and can stoichiometrically react with equivalent amounts of ^•^NO [30]. This reaction leads to the in vivo formation of ^•^NO–hemoglobin deoxygenation end-products (methemoglobin, nitrate) and iron–nitrosylhemoglobin complexes, thus contributing to decrease ^•^NO bioavailability [30].

### 2.3. Heme and Iron Release

Under mild to moderate hemolysis, Hb is bound in plasma by haptoglobin (Hp) forming a complex, which prevents the release of free iron while continuing to maintain the ability to bind ^•^NO [31]. The complex is internalized and degraded through the CD163 receptor found on macrophages and CD91 receptors found on hepatocytes [32]. The release of heme from HbS is faster than from normal Hb [29]. Its characteristic hydrophobicity allows heme to intercalate into the cell membranes and magnify the intracellular heme-dependent reactive oxidizing species generation. In addition, under inflammatory condition, O_2_^•−^ and H_2_O_2_, released by activated cells, can react with heme and catalyze both the non-enzymatic generation of reactive oxidizing species as well as the release of free reducing ferrous ions, which in turn may increase the Fenton-driven reactions and induce further oxidative and nitrosative stress [33] (Figure 1). These events amplify the formation of ROS inside cells leading to additional damage to intracellular components including proteins, lipids, and DNA [34,35]. Consequently, fundamental functions of cells may be compromised by this heme- and iron-mediated increase of ROS formation, such as the intracellular signaling mediated by oxidant-sensitive targets, the expression of pro-inflammatory transcription factors, the integrity of membrane channels, the activity of metabolic enzymes, inducing finally cell apoptosis and death. In addition, heme-derived oxidants induce recruitment of leukocytes, platelets and RBCs to the vessel wall; produce lipoproteins oxidation and consume ^•^NO in the formation of strong oxidants such as ONOO^−^ (Figure 1) [36].

### 2.4. Decreased ^•^NO Bioavailability

In SCD, all ^•^NO biological functions, including the regulation of vascular tone, the control of cell activation, aggregation and adhesion in the vascular compartment, are compromised so that vasoconstriction, pulmonary hypertension, endothelial dysfunction, thrombosis and inflammation characterize the vasculopathy linked to this disease [39,40,41]. In the absence of interactions with biological targets, ^•^NO reacts with oxygen (9 × 10^7^ M^−1^s^−1^) to form nitrite. In biological systems, this reaction is slower with respect to those occurring with metal-containing macromolecules generating either nitrate, as in the case of hemoglobin (6–8 × 10^7^ M^−1^s^−1^) and nitrite in the case of ceruloplasmin acting as a ^•^NO oxidase and nitrite synthase [42]. Nitrite is not an inert end-product, but it has bioactivity since it can generate ^•^NO from its reaction with metalloproteins, as in the case of HbS itself, which has been reported to possess nitrite reductase activity [43]. In addition, the concentration of these ^•^NO-derived metabolites is deeply affected by the dietary intake and renal function. In plasma of SCD patients, the concentrations of nitrite and nitrate appear to undergo modifications, i.e., in the steady state of disease they have been found to be comparable to those measured in normal volunteers, but they decreased with acute pain and acute chest syndrome [44,45]. The impairment of ^•^NO availability in SCD is mainly linked to the intravascular hemolysis (Figure 1). Cell-free hemoglobin has indeed a large impact on the bioavailability of ^•^NO. While the reaction of ^•^NO with oxygenated Hb results in methemoglobin and nitrate formation, its binding to deoxygenated hemoglobin favors the formation of a stable FeIIHb-NO complex, which can be involved in Fenton reactions [36]. Interestingly, a gender difference was also described in ^•^NO availability in SCD patients. In particular, thanks to the protective effects of estrogen on the expression and activity of NOS, women are more protected from the decrease of ^•^NO availability [46].

Another crucial metabolite contributing to vascular impairment in SCD is L-arginine. Besides being used by NOS to generate ^•^NO, L-arginine is also the substrate of the enzyme arginase, which competes with NOS for L-arginine, generating urea and ornithine. Since the arginase activity is significantly high in RBCs compared to plasma [47], the hemolysis causes the release of the enzyme from RBCs into plasma allowing to the rapid consumption of L-arginine, reducing the substrate for ^•^NO synthesis and consequently its bioavailability in SCD (Figure 1) [48]. In SCD patients the L-arginine supplementation increases both the nitrite plasma concentration as well as the HbF synthesis, suggesting the beneficial effects of ^•^NO on the erythroid progenitor cells [49].

Finally, ^•^NO-derived metabolite nitrite is also consumed by the heme-containing myeloperoxidase (MPO). This enzyme, localized within neutrophils and released upon cell activation, catalytically reacts with nitrite in the presence of H_2_O_2_, generating powerful radical intermediates, such as nitrogen dioxide (^•^NO_2_), which can oxidize and nitrate protein tyrosine residues [50]. MPO also could contribute to the pulmonary hypertension and acute chest syndrome in SCD, since elevated MPO immunoreactivity has been measured in the alveolar epithelium of lung tissues from patients with SCD [51].

### 2.5. Uncoupling of NOS Activity

Under normal conditions, the oxygenase and reductase subunits of NOS dimerize in the presence of O_2_ and the cofactors NADPH and tetrahydrobiopterin (BH4), allowing to convert L-arginine into ^•^NO and L-citrulline. BH4 and L-arginine maintain the enzyme as a dimer and thus are key regulators for NOS activity, so that when their concentration is low, the enzyme dissociates into monomers generating O_2_^•−^ instead of ^•^NO, a phenomenon, which has been named “NOS uncoupling”. NOS uncoupling and generation of O_2_^•−^ may be favored in SCD, i.e., when hemolysis determines reductions of the concentrations of L-arginine [22] and BH4 due to its oxidation mediated in particular by ONOO^-^ abundantly produced in SCD (Figure 1) [22,52]. This condition further reduces ^•^NO bioavailability in both SCD and other vasculopathies [53]. Loss of ^•^NO bioactivity and oxidative stress increases the risk for further vessel occlusion by pro-inflammatory effects that render endothelial cells more adhesive and chemo-attractive for circulating leukocytes, promoting thrombotic mechanisms.

### 2.6. Increased Asymmetric Dimethylarginine

Asymmetric dimethylarginine (ADMA), a methyl derivate of the amino acid arginine, is produced by the physiological degradation of methylated proteins. It is continuously produced in various cell types, including endothelial cells. ADMA is the major endogenous inhibitor of NOS competing with the enzyme’s natural substrate L-arginine for binding at the active site of the enzyme. In addition, ADMA boosts all NOS isoforms to uncouple, by converting them from ^•^NO-producing enzymes to enzymes generating O_2_^•−^ and the derived oxidants (i.e., H_2_O_2_, ^•^OH) [54,55], contributing to increase oxidative stress and decrease the ^•^NO bioavailability. Elevated plasma concentrations of ADMA represent a novel risk factor for the development of endothelial dysfunction and a predictor for all-cause and cardiovascular mortality. Increased plasma ADMA levels mainly occur following inhibition of the enzyme responsible for ADMA catabolism, dimethylarginine dimethyl-amino-hydrolase (DDAH), by oxidative stress triggered by several cardiovascular risk factors. Increased plasma levels of ADMA have been found in SCD patients that further limiting ^•^NO bioavailability, and these increased plasma levels were associated with increased amounts of soluble vascular cell adhesion molecule-1 (sVCAM-1), a marker of endothelial activation normally suppressed by ^•^NO [56]. In SCD patients, ADMA is also associated with hemoglobin levels suggesting a correlation with more severe intravascular hemolysis. Moreover, high ADMA levels in plasma may be associated with SCD-related pulmonary hypertension and early death [56,57]. Indeed, high plasma ADMA levels are correlated with the hemolytic markers and may represent a risk factor for high tricuspid regurgitated jet velocity in children with SCD [58].

## 3. Oxidative Damage to Intracellular Components in SCD RBCs

As reported above, the common denominator in the damaged SCD RBCs is the increased HbS auto-oxidation and the Fenton chemistry driven by denatured heme bound to the RBC membrane, further the amplified by the oxidants generated by the increased activity of ROS-generating enzymes.

The repeated HbS polymerization and depolymerization causes increased ROS generation [40,41]. In addition, HbS polymerization, linked to the oxygenation/deoxygenation cycles, induces conformational change in the molecule leading to the formation of HbS polymers (Figure 1). These polymers boost oxidative stress and, growing rapidly to form long fibers, increase cellular rigidity, deformability and alter cell membrane leading to RBC sickling, cellular energetic failure, impaired rheology, and hemolytic anemia [59].

A significant ROS production within sickle RBCs is mediated enzymatically by NADPH oxidase and stimulated by plasma signaling factors [24]. In particular, ROS derived by NADPH oxidase may cause direct oxidative damage to a variety of subcellular structures, reducing erythrocyte deformability and resulting in increased RBC fragility and hemolysis [24]. NADPH oxidase activity may deplete the cellular pool of NADPH, thus impairing the ability of the RBC to maintain its antioxidant defenses. In RBCs from SCD patients, oxidative stress has an effect on cytoskeletal proteins as well as on cell membrane. ROS produced at the plasma membrane level indeed are not readily scavenged by the cytoplasmic antioxidant system [60] and can boost further oxidative damage to membrane lipids and proteins [61].

### 3.1. Oxidative Damage to SCD RBCs Cytoskeletal Proteins

Due to their chemical properties, oxidizing species directly react with targets molecules inducing reversible or irreversible post-translational protein modifications (PTPM) in specific amino acid residues, such as cysteine, tyrosine, tryptophan and methionine. These PTPM are of particular importance when generated in molecules involved in intracellular signaling since they can affect cell functions and fate. Reversible PTPM, oxidation (sulfenylation, nitrosylation, glutathionylation, sulfhydration, sulfenic acid and disulphides formation) is indeed the basis of the thiol-mediated redox-based signaling, while the irreversible PTPM oxidation (carbonylation, nitration, sulfonic acid formation) allows in most cases functional decline of the target proteins.

Two others key PTPM are phosphorylation and ubiquitination. The phosphorylation is the most common reversible PTPM regulating the intracellular signaling dependent on enzymes and receptors, which undergo to continuous phosphorylation and dephosphorylation events. Less investigated than phosphorylation, ubiquitination is an ATP-dependent irreversible PTMP of a target molecule consisting in the covalent binding of ubiquitin mediated by the selective proteolytic system (the ubiquitin proteasome system), active in human RBCs and found to be associated with protein oxidation and aggregation [62].

The cytoskeleton of RBCs is a dynamic and complex structure. Its assembly and integrity are fundamental pre-requisites for the survival and deformability during their transit through capillary networks. Cytoskeleton lies under the lipid bilayer of the cell membrane and consists of α and β spectrin heterodimers anchored to the lipid bilayer by the band 3/ankyrin protein complex [63]. Spectrin is a major target of reactive oxidizing species and its oxidation results in disruption of its interaction with actin and protein 4.1 [63].

In RBCs from SCD patients, several proteins undergo oxidative-mediated PTPM, including HbS itself and several cytoskeletal proteins. Moreover, revealed in RBCs from transgenic SCD mice, these irreversible PTPM detected in HbS were found in β chain and included the irreversible oxidation of Cys93 and the ubiquitination of Lys96 and Lys145 [64,65]. HbS ubiquitination has been attributed to the accumulation of oxidatively damaged HbS molecules in RBCs as well as in MP and could be due to the likely redox imbalance-dependent proteasomal inhibition in SCD [64]. A recent analysis of RBCs and MP proteome showed, in addition to higher amounts of protein carbonyl groups, increased phosphorylation and ubiquitination of cytoskeletal proteins from patients SCD with respect to control cells [65]. Interestingly, these PTPM have been identified in band 3, spectrin, ankyrin, carbonic anhydrase, and band 4.1 and were significantly reduced after the treatment in vivo with hydroxyurea [65].

Undoubtedly, the tyrosine phosphorylation of band 3 is a key mediator in the events leading to intravascular hemolysis and MP release, hesitating in vasoocclusion in SCD [66]. In RBCs, band 3 tyrosine phosphorylation is regulated by the activity of phosphotyrosine kinase (p72Syk) which phosphorylates the protein and phosphotyrosine phosphatase engaged to dephosphorylate it. Being a redox sensor, the activity of tyrosine phosphatase is inhibited under oxidative stress conditions favored by HbS autoxidation, and the consequent up-regulation of band 3 tyrosine phosphorylation favors the dissociation of band 3 from the other cytoskeletal proteins (spectrin and actin), altering the RBSs morphology [66]. Interestingly, in RBCs from SCD patients, the degree of band 3 tyrosine phosphorylation correlated with the amount of intracellular HbS of cell-free HbS and RBC-derived MPs in blood plasma. Importantly, the inhibition of the phosphotyrosine kinase inhibited Band 3 tyrosine phosphorylation preventing the amounts of cell-free Hb and the release of RBC-derived MPs [66].

The increased HbS autoxidation in SCD is known indeed to favor the formation of metHb oxidation products (hemichromes) and heme loss, as well as the irreversible protein denaturation and precipitation as Heinz bodies.

Hemichromes have a strong affinity for cytoplasmic domain of band 3 and, following their binding, lead to band 3 oxidation and clusterization.

Moreover, in other hemolytic disorders such as thalassemia, it has been demonstrated that hemichromes phosphorylate the band 3 protein, inducing membrane instability and release of micro-particles having an increased thrombotic risk in hemolytic disorders [67].

Moreover, an increase of ROS production by auto-oxidized HbS can increase the accumulation of oxidative lesions by membrane components (Figure 1). In particular, they can degrade polyunsaturated lipids forming malondialdehyde as a by-product and damage proteins localized in the region near membrane-associated hemoglobin [68]. HbS has a higher affinity for band 3 than normal Hb [69] and hemichromes derived from the autoxidation of membrane-associated HbS consistently establish a disulfide bond with the cytoplasmic domain of band 3. This binding activates the tyrosine kinase-linked RBCs intracellular signaling leading to the phosphorylation of band 3 cytoplasmic domain, to band 3 clusterization and to the rupture of the binding with the other cytoskeletal protein ankyrin. Reactive oxidizing species production can directly oxidize and induce conformational modifications of the membrane protein band 3 and abnormal exposure of phosphatidylserine (PS), considered marker of RBC senescence. The band 3 is the main RBC membrane protein essential for ensuring structural cytoskeletal organization. It is an important candidate to participate in RBC-vascular endothelium interaction. Blebs and micro-particles (MPs) containing HbS-derived oxidation products, such as metHb, heme and its derived oxidation products (hemin) as well as free iron, accumulate in there and are then released in the vasculature from RBCs membrane (Figure 1) [70,71]. This event produces three significant consequences: (i) inhibition of the adherence of sickle cells to endothelium, (ii) occurrence of binding sites for natural band 3 antibodies (IgG class) that are able to react with the complement system, and (iii) boosting of increase oxidative stress and vasculopathy associated to SCD. Importantly, the recognition by complement system leads to elimination of the labeled cells by macrophages in the spleen [72]. Plasma from SCD patients is rich in RBC-derived MPs, which contain heme and express PS [73]. It has been demonstrated that RBC membrane alterations in SCD enhance the activation of acid sphingomyelinase, contributing to RBC-derived MPs generation. In addition, the circulating heme-overloaded MPs can in this manner transfer the pro-oxidant heme potential to the vasculature contributing to increase oxidative stress-mediated pathophysiology of vascular dysfunction in SCD [73,74] (Figure 1). MPs can be also internalized by myeloid cells and promote pro-inflammatory cytokine secretion and endothelial cell adhesion [74,75].

### 3.2. Oxidative Damage to SCD RBCs Membrane Lipids

In addition to protein components, the RBC membrane is composed by a lipid bilayer consisting of a highly complex and dynamic system where lipids are continuously renewed. The lipids can move across the bilayer always providing the suitable environment for the membrane proteins and maintaining the proper barrier separating the intracellular compartment from the extracellular one. Mature RBC lipid bilayer is composed mainly by (i) phospholipids, i.e., phosphatidylcholine (PC), phosphatidylethanolamine (PE) and PS; (ii) sphingomyelin (SM); and (iii) cholesterol. In RBCs these lipid species are highly organized in asymmetric specific phospholipids (micro-domains). As an example, PS is exclusively in the inner leaflet of the bilayer and the PC and SM mainly in the outer leaflet. These micro-domains maintain highly conserved interactions with the membrane proteins focused to regulate the traffic of ions and signals across the bilayer. In general, the oxidative reactions occurring on lipids can be induced through (i) a radical-dependent mechanism, involving oxidative intermediates derived from O_2_^•−^, ^•^NO or ^•^OH radical generated by the metal-catalyzed H_2_O_2_ reactions; (ii) a non-radical, non-enzymatic-dependent mechanism, involving species such as HOCl, singlet O_2_ and ozone (O_3_); and (iii) a enzymatic-dependent mechanism, involving enzymes such as lipoxygenase and cyclooxygenase [76]. As a function of the involved oxidant species, the lipid oxidation/peroxidation products include simple hydroxy fatty acids, oxidized cholesterol species, isoprostanes, nitro-fatty acids and lipid aldehydes [76].

In SCD RBCs, the main mechanism involved in lipid oxidation is mediated by formation of ROS and heme-dependent intermediates. The presence of auto-oxidizing HbS in the membrane and the high rate of intracellular ROS production result in the oxidative damage to membrane lipids, to the loss of membrane lipid asymmetry resulting in altered membrane surface properties and permeability as well as to PS exposure [77]. PS externalization, linked to a Ca_2_^+^-dependent and thiol-mediated mechanisms involving enzymes such as flippase and scramblase in SCD RBCs [78], is a critical event in the disease progression. In SCD indeed, spleen activity to remove these PS-exposing RBCs is compromised, and this explains the high numbers of these cells in the SCD patients circulation [77]. PS externalization is considered a hallmark of premature cell aging favoring the removal of RBCs from the circulation, contributing to the occurrence of anemia as well as of vaso occlusion and endothelial dysfunction as a consequence of the interactions of PS-exposing SCD RBCs with platelets and vascular endothelium [79].

Moreover, SCD is characterized by increased plasma levels of secretory phospholipase A2 (sPLA2), a potent inflammatory mediator able of selectively hydrolyze phospholipids in RBCs exposing PS, promoting their hemolysis [80,81]. Activation of sPLA2, by phospholipid hydrolysis, also generates phospholipid breakdown products affecting endothelial function [81]. Moreover, PS-exposing RBCs show the activation of a phospholipase D, which catalyzes the hydrolysis of PC into phosphatidic acid and choline [82]. Phosphatidic acid undergoes degradation generating lysophosphatidic acid, a bioactive lipid important in a multitude of cellular processes such as inflammation, vascular dysfunction and migration [81]. In patients with SCD, the final modifications of RBCs mediated by the products of lipid peroxidation consist of alterations of the interactions of membrane lipids with plasmamembrane and cytoskeletal proteins causing the loss of cell membrane integrity and the red blood cell hemolysis (Figure 1).

## 4. RBC Hemolysis

There are two clinical types of hemolysis in SCD: (i) extravascular hemolysis, which occurs when aged or defective RBCs are removed from macrophages by phagocytosis (generally, this does not involve the release of hemoglobin into the plasma compartment) [23] and (ii) intravascular hemolysis, which occurs when hemoglobin is de-compartmentalized and released into plasma, where it scavenges ^•^NO [83,84]. In extravascular hemolysis, RBCs are phagocytized by macrophages with (i) increased PS exposure on the outer leaflet of the membrane (a senescence signal); (ii) increased surface-bound immunoglobulin; and (iii) membrane “pits” and “pocks” caused by precipitated denatured hemoglobin [83]. Following intravascular hemolysis, RBCs release into the plasma danger-associated molecular pattern molecules (DAMPs) that impair endothelial function and drive oxidative and inflammatory stress, leading to chronic vasculopathy and pulmonary hypertension [84]. eDAMPs are represented by circulating hemoglobin and heme. Free hemoglobin scavenges ^•^NO, reducing its bioavailability, favoring ROS production and causing oxidative ^•^NO consumption [85]. Heme especially activates innate immune sterile inflammation pathways through the toll-like receptor system 4 (TLR4) and NALP inflammasome signaling [86] (Figure 1). The binding of free heme to receptors present in cell membranes can deeply affects the intracellular signaling through the modulation of oxidant-sensitive cellular pathways including growth factor receptors, kinases, and transcription factors, which in turn drive the cell fate toward to apoptosis and cell death [82]. In ECs, the binding of heme to Toll-like receptor-4 (TLR4) leads to NF-κB signaling pathway activation [87] playing a role in the initiation of inflammation by (i) up-regulating pro-inflammatory genes; (ii) increasing the release of highly pro-inflammatory cytokines, such as interleukin-1β (IL-1β), IL-18, and IL-6; (iii) modifying the intracellular metabolism; and (iv) favoring the expression of adhesion molecules, such as VCAM-1, ICAM-1, E-selectin and P-selectin, that are all markers of endothelial dysfunction and function as receptors for leukocyte adhesive [36,88,89,90] (Figure 1). Free heme and iron also promote inflammatory injury via activation of innate immune responses in macrophages and monocytes [91]. As reported for heme, also the heme-derived oxoferryl contributes to the pathogenesis of hemolytic disorders by acting as a potent pro-inflammatory agent. Moreover, it has been reported that oxoferryl induces the rearrangement of cytoskeletal proteins, such as actin, to form intercellular gaps, to disrupt the integrity of ECs, to induce the activation of NF-kB and the expression of adhesion molecules (E-selectin, ICAM-1, and VCAM-1) in ECs [92]. All these events contribute to boost inflammatory conditions, vaso occlusion and subsequent decrease of tissue oxygenation [93] (Figure 1).

## 5. Cell Adhesion and Vessel Occlusion

Oxidative stress is undoubtedly the main cause of chronic vasculopathy characterizing SCD. Vascular lumen obstruction in SCD results from interaction of pro-oxidant RBCs, activated leukocytes and platelets and the vessel wall. The decreased ^•^NO availability, the oxidant-mediated activation of the coagulation system including platelets and plasmatic coagulation factors, and the oxidant-driven release of MPs cooperate to the outcome of vessel occlusion. The microvasculature is a major target in SCD, and the activation of endothelial cells is a critical component of the microvascular responses accompanying this disease [59]. Pro-oxidant sickle RBCs are more adhesives and can bind to endothelial cells, sub-endothelial matrix proteins, plasma proteins, leukocytes, platelets, and other RBCs, than normal RBCs. Adhesion molecules such as α4β1 integrin (also known as VLA-4) and CD36 are overexpressed and mediate the adhesion to the endothelium. α4β1 RBC binds to endothelial VCAM-1 and fibronectin, a component of the sub-endothelial cellular matrix that comprises the endothelial basement membrane. CD36 mediates adhesion through a thrombospondin (TSP) bridge to αVβ3, an integrin expressed by activated microvascular and large vessel endothelium. SCD patients have elevated plasma levels of soluble TSP, and it has also been shown that it mediates sickle RBC adhesion to the blood vessel wall by CD47, an integrin-associated protein expressed on both normal and HbS RBCs [39]. Moreover, it has been reported that RBCs bind αV β3 by PS and laminin by Lutheran/basal cell adhesion molecule (Lu/BCAM) proteins [94].

Leukocytes (lymphocytes, neutrophils, and monocytes) play a critical role in SCD by adhering to and stimulating the vascular endothelium and aggregating with pro-oxidant RBCs and activate platelets (Figure 1). The leukocytes most frequently involved in the process of adherence to vessel walls are the neutrophils. The adhesion molecules that mediate adherence of leukocytes to vascular endothelium include α2Lβ2 integrin (CD11a/CD18 heterodimer), αMβ2 integrin (CD11b/CD18 heterodimer), CD31 or platelet-endothelial cell adhesion molecule-1, the CD36, leukocyte selectin (L-selectin or CD62L), and platelet selectin glycoprotein ligand-1 (PSGL-1 or CD162), which is also expressed on vessel endothelial cells. Their ligands on vascular endothelium are ICAMs-1,2,3 [95]. In addition to adhering to vascular endothelium, leukocytes bind platelets and RBCs to form cell aggregates that could obstruct the lumen of small blood vessels more effectively than single cells (Figure 1).

## 6. Advanced Glycoxidation and Lipoxidation End-Products

Advanced glycoxidation end-products (AGEs) and advanced lipoxidation end-products (ALEs) have been reported to play an important role in the development and progression of diseases such as, diabetes [96], chronic renal diseases [97], cardiovascular diseases [98] and neurological disorders [99], that have as hallmark oxidative stress. AGEs and ALEs, present mainly in heated food, are generated either endogenously or exogenously through different mechanisms by heterogeneous precursors. Although they are not only well established markers of oxidative stress, AGEs and ALEs show themselves pro-oxidant activity and ability to affect intracellular signaling [100,101].

AGEs are a class of macromolecules generated through oxidative and non-oxidative reactions (e.g., the Maillard reaction) between reducing sugars and their oxidation products (ketoaldehydes, deoxyosones and ketoamines) with nucleophilic amino groups of aminoacyl residues in proteins [99]. AGEs include Nε-(carboxymethyl)lysine (CML) and Nε-(carboxyethyl)lysine (CEL), which are the adducts of the small reactive aldehydes glyoxal and methylglyoxal, respectively, with lysyl resides of proteins. Because CML and CEL may derive from free-radical attack to derivatives of both carbohydrate and lipid metabolism, as already mentioned in paragraph 2.3, they are referred to as mixed AGEs/ALEs [100].

Examples of endogenously produced AGEs are glyoxal, methylglyoxal and its derivatives (generated by the degradation of the triose phosphate intermediates originating from the glycolytic processes through non-enzymatic or lipid-mediated enzymatic reactions involving cytochrome P450 2E1, myeloperoxidase and amino oxidase), as well as 3-deoxyglucosone and its derivatives [101]. Some compounds included in AGEs group, such as the carboxymethyl-lysine, have the same structure of ALEs since they are generated from the common precursors, in this case being glyoxal or acrolein formed by both lipid and sugar oxidative degradation pathways [102].

The mechanisms at the basis of AGEs damaging include: (i) alteration of protein function via modification of cysteinyl, lysyl and arginyl residues and via intra- and intermolecular cross-linking involving arginyl and lysyl residues; (ii) interference with matrix-matrix and/or matrix-cell interactions like collagen and laminin, causing thickening of membranes and luminal narrowing of blood vessels; (iii) receptor-mediated pathologic gene expression; and (iv) boosting of oxidative stress. [103,104,105]. Through interaction with their receptors (RAGE), transmembrane proteins that are part of the immunoglobulin superfamily expressed on different cell types including endothelial cells, AGEs stimulate the intracellular signaling pathways boosting the formation of pro-inflammatory cytokines, adhesion molecules (through activation of NF-kB) and the production of ROS, such as O_2_^•−^ through the activation of NADPH oxidase activity [106]. AGEs, produced on RBC membranes, bind to specific AGE receptors on ECs and can: (i) alter gene expression in these cells [107]; (ii) modify the production of thrombomodulin (tissue factor and adhesion molecule of vascular cells-1) and (iii) favoring pro-coagulant changes that increase RBC adhesion to the vessel wall [105].

ALEs include several compounds generated in the course of oxidative stress via free radical-initiated chain reactions of lipid peroxidation. These include a number of highly reactive aldehydes, such as acrolein, malondialdehyde (MDA), 4-hydroxy-2-nonenal (HNE), 4-hydroxy-2-hexenal (HHE), nonenal, 4-oxo-2-nonenal (ONE), all derived from the peroxidation of PUFAs, either free or as components of membrane phospholipids, as well as certain isoprostanes deriving from the endocyclization of hydroxyperoxyl radicals and some derivatives of leukotriene and prostaglandin metabolism. These are all able to modify covalently proteins, DNA and aminophospholipids by nucleophilic attack, thus damaging seriously cell functions and viability [100,101,102,103,107,108].

Nuclear and mitochondrial DNA are key targets of ALEs reactions. Guanine, adenosine and cytosine are the most modified bases, with guanosine being most reactive due to its high nucleophilicity [100,109]. Interestingly, reactive carbonyl species can also activate an intracellular antioxidant response, which prevents their accumulation and toxicity. This involves the activation of the factor that favors the transcription of genes linked to antioxidants, such as enzymes linked to the synthesis of glutathione, hemeoxygenase, Prx, SOD, thioredoxin reductase and thioredoxins [100]. The formation of adducts of the products of lipid peroxidation (LPO) products and of protein–protein cross-links mostly affects histidyl, lysyl and cysteinyl residues of proteins, leading to conformational changes, denaturation, oligomerization and inactivation, including the loss of enzymatic activity. Among the different targets of lipoxidation there are cytoskeletal proteins (actin, laminae), histones (H2A), glycolytic enzymes (glyceraldehyde-3 phosphate dehydrogenase, pyruvate kinase), molecules involved in cell signaling and transcription (H-RAS, KEAP-1, HSP70 and HSP90) [108]. Protein/enzyme lipoxidation affects their structure and function leading to the modification of cell homeostasis, gene expression, cell signaling and fate [108].

### AGEs and ALEs in SCD

High levels of AGEs have been measured in both blood plasma and RBCs of SCD patients. [110,111]. High levels of Nε-(carboxymethyl)lysine (CML), Nε-(carboxyethyl)lysine (CEL) and pentosidine were detected in plasma and in RBCs from patients with SCD [108,109], as shown in Table 1 [110,111]. In addition, also the rate of AGEs accumulation in RBCs was higher in SCD patients, suggesting intracellular/extracellular SCD-related conditions favoring AGE synthesis [110]. Interestingly, the correlation between the plasmatic concentration of two AGEs (pentosidine and Nε-(carboxymethyl)lysine) and hemolysis, hemolysis-related complications and the hemolytic rate during the clinically asymptomatic state suggested that these compounds might be involved in the hemolytic pathophysiology of SCD [111].

Compared with healthy controls, blood plasma from SCD patients also contains high concentration of the soluble form of RAGE, which has been proposed as a biomarker of vasculopathy in SCD [112]. The mechanisms hypothesized contributing to the release of this soluble form of RAGE are (i) a AGEs-mediated and increased activity of matrix metalloproteinase-9 [113,114], which proteolytical cleaves the cell surface full-length RAGE able in turn to promote further RAGE release in a reactive oxidizing species-dependent manner [115] and (ii) an oxidative stress-mediated modification of splicing mechanisms in ECs, which directly could affect the RAGE levels [116]. The soluble form of RAGE might be of particular importance to identify at-risk patients and direct them a treatment with anti-RAGE antibodies that inhibit the activation of signaling downstream to these AGE receptors.

Although lipid peroxidation is elevated in tissues and represent an important source/biomarker of oxidative stress [117,118,119], the specific presence of ALEs in fluids and tissues of SCD patients has been currently not investigated in depth, with the exception of the measurement of Nε-(carboxymethyl)lysine (CML) and Nε-(carboxyethyl)lysine (CEL) [110] (Table 1). Other biomarkers of oxidative stress, which were investigated in SCD are malondialdehyde (MDA), which derives from the decomposition of certain products of the peroxidation of PUFAs (albeit not only from them) and F2-isoprostanes, which are the products of endocyclization of hydroxyperoxyl radicals generated in the course of radical-initiated lipid peroxidative chain reactions (Table 1). F2-isoprostanes levels have been hypothesized to correlate with the patient’s clinical status, since their concentration was increased in plasma of SCD subjects during the acute chest syndrome as compared with normal volunteers and significantly declined in post-exchange transfusion to a level similar to that of patients with SCD at baseline [119]. Other studies report that F2-isoprostanes levels were not modified in SCD patients with respect to healthy subjects during the period of relative health [120] and that they were increased in steady-state HbS patients [121].

The MDA level was significantly higher in SCD patients compared to the control group [122,123,124,125]. Interestingly, MDA formation was significantly increased in LDL and HDL purified from patients with SCD and correlated with increased Hb and total plasma heme levels [123]. It well established that LDL oxidation plays a key role as in atherogenesis [126] and is triggered by reactive oxidizing species or by the uptake of heme [27]. The highly hydrophobic LDL competes indeed with plasma heme-binding proteins, such as hemopexin (Hpx) and albumin, for the free heme, so that about 80% of heme added to plasma is immediately taken up by lipoproteins [27]. The LDL oxidative reactions lead to the formation of lipid hydroperoxides and other oxidants able to further amplify the oxidative degradation of the heme and the release of heme iron. It is hopeful that further studies will clarify the mechanisms of formation and the role AGEs and ALEs in the etiology and progression of SCD and that these studies could suggest therapeutic potential of interventions targeting upstream the formation of both compounds aimed to turn off an important source of damage in SCD.

**Table 1:** Oxidative modifications of red blood cell (RBC) and plasma proteins detected in patients with SCD.

Compound	Specificity in SCD	Tissue	References
Advanced lipoxidation end-products (ALEs) (adducts with oxylipin carbonyl compounds)			
F2-isoprostanes adducts	Biomarker of lipid peroxidation	Plasma	[119,120,121]
Malondialdehyde (MDA) adducts	Biomarker of lipid peroxidation	Plasma	[122,124,125]
Mixed advanced lipoxidation end-products (ALEs)/advanced glycoxidation end-products (AGEs)			
Nε-carboxymethyllysine (CML)	Biomarker of hemolysis-related organ complications and vascular pathology	Red blood cells, plasma	[110,111]
Nε-carboxyethyllysine (CEL)	Biomarker of hemolysis-related organ complications and vascular pathology	Red blood cells, plasma	[110,111]
Adducts generated by the Maillard reaction			
Pentosidine *	Biomarker of hemolysis-related organ complications and vascular pathology	Red blood cells, plasma	[111]

* Pentosidine is produced by the reaction of nucleophilic amino groups of aminoacyl residues in proteins with the carbonyl group of ribose in the Maillard reaction. It is able to determine the formation of cross-links between arginine and lysine residues and is commonly exploited as a biomarker of AGEs formation.

## 7. Antioxidant Defenses in SCD

It is hypothesized that in SCD tissues the antioxidant system is depleted due to the pro-oxidant status (Figure 1). This is also true for SCD RBCs in which there is a decreased concentration of both low molecular weight antioxidants such as vitamins A, C and E [29] and the activity/expression of some high molecular-weight antioxidant enzymes, such as SOD [19,45,127], Cat [19,45,128], GR [127] and Gpx [125,128]. However, in the case of SCD, several studies also reported an increased activity/expression of these antioxidant enzymes (Figure 1) [10,28,127,129,130]. This could be a defense mechanism linked to the accumulation of O_2_^•−^ and H_2_O_2_ in red blood cells and SCD tissues. In addition to SOD and Cat, RBCs contain a strategic H_2_O_2_-detoxifying system, which play a key role in maintaining the suitable thiol/disulfide intracellular equilibrium (sulfenic acids, S-nitrosothiols, S-glutathionylation, etc.) linked to redox signaling [131,132]. This antioxidant system includes the couple GSH and its oxidized form (GSSG), GR, GPx, glutharedoxin (Grx), the couple thioredoxin (Trx)/thioredoxin (TrxR), peroxiredoxin (Prx), and the redox couple NADP+/NADPH [131,132].

GSH is the principal non-protein low molecular-weight compound highly concentrated in human tissues (up to 10 mM) and is the major cellular redox buffer. GSH directly reacts with several oxidizing species, including ^•^OH, ferrylHb, HClO, and ONOO^−^, and is the substrate of GPx, which catalyzes the conversion of H_2_O_2_ to water. In these reactions GSH is oxidized to GSSG, and the ratio GSH/GSSG is commonly used as an indicator of the cellular redox environment. In addition to the enzymatic de novo synthesis, GSH intracellular content can be regulated by (i) reduction of GSSG, mediated by the NADPH-dependent GR activity; (ii) PPP oxidative branch or (iii) protein glutathionylation and de-glutathionylation reactions, which promote the formation of mixed disulfides formed by GSH with redox-sensitive Cys residues of proteins or the release of GSH from pre-formed mixed disulfides, respectively [131,132].

Prx, Trx/TrxR system and Grx also contribute to cellular protection by reducing oxidized critical thiols in key enzymes/proteins and maintaining the suitable intracellular redox state. In addition, the isoform 2 of Prx (Prx2), the third most abundant protein in RBCs, competes effectively with Cat and GPx to scavenge low levels of H_2_O_2_ [133]. GPx and Prx are regenerated by the GSH/GR and Trx/TrxR systems. The correct activity of these antioxidant systems bases on the redox couple NADP+/NADPH, which is crucial for the intracellular redox homeostasis providing reducing equivalents for the above-described detoxifying enzyme systems, i.e., GR/GPx/GSH and TrxR/Trx/Prx [131,132].

Contradictory results have been reported with regards the NADPH measurement in RBCs of SCD patients. Indeed, NADPH concentration was found to be both not different [120] and decreased [134] or increased [128] with respect to control patients. These results could reflect different SCD-unrelated clinical states of the patients, such as inflammatory conditions in which for example the increased NADPH oxidase activity could contribute to deplete the cellular pool of NADPH [24,28,135], or the establishment of unappreciated compensatory intracellular mechanisms.

The analysis of glutathione content indicated that both GSH and GSSG concentration was significantly decreased SCD patients [136,137,138]. Quantitatively, the GSH concentration measured in RBCs and in plasma of SCD patients was about 36% [28] and 25% [18] lower, respectively, compared to healthy controls, despite sufficient availability of its precursors (glutamate, cysteine and glycine) in both plasma and within the RBCs [136]. The lower GSH concentration in SCD patients has been explained with the increase of thiol consumption, hypothesis confirmed by the measurement of the increase of the rate of GSH synthesis (higher by about 57% in RCBs of SCD patients with respect to healthy subjects) [136,139]. In further support of this hypothesis, the concentration of GSH precursors (glutamine, cysteine and glycine) was increased, and the amount of glutamate (the precursor of glutamine) was decreased in both plasma and RBCs of SCD patients [136]. Moreover, the concentration of NADPH (produced when glutamine is metabolized to glutamate) was increased with respect to healthy subjects [134]. In addition to GSH, also GSSG levels were lower in RBCs of patients with SCD compared to controls [28]. The hypothesis proposed to explain the decrease of both GSH and GSSG is the formation of protein–SG mixed disulfides [28]. Thiol groups of several proteins may indeed undergo to this post-transcriptional modification, called glutathionylation, which plays a critical role in redox signal transduction by binding proteins/enzymes and reducing or increasing their functions [140]. The process depends on GSH and GSSG concentration and is a radical-mediated process. In SCD RBCs, HbS can undergo to glutathionylation, which increases the oxygen affinity, reduces the heme-heme interactions and inhibits the sickling protein [141].

Prx2 was found to be significantly impaired within SCD RBCs [28,128,130], with about 18% inactivation as result of oxidation to active thiol group to sulfinic acid, whereas the inactive enzyme was virtually undetectable in control cells [130]. Indeed, increased binding of Prx2 to the RBCs membrane was detected in dense SCD RBCs [142]. Finally, a significant content of the Prx2 dimeric form has been detected in the cytosol so that it has been proposed as a hallmark of oxidative stress inside SCD RBCs [143]. In fact, under oxidative stress conditions leading to exceedingly elevated H_2_O_2_ concentrations, Prx2 could persist into the oxidized/dimeric form, which lack to scavenge H_2_O_2_, dissociate from RBCs membrane, and the switch to other signaling- or regulatory-linked roles, such as Ca_2_^+^-activated-K^+^ transport or chaperone activity [144,145]. Finally, the ability of Prx2 to bind to Hb has been hypothesized to be crucial for stabilizing the hemoprotein, protecting it from the excessive oxidative stress. RBCs form SCD patients indeed showed a reduced binding of Prx2 to Hb making the hemoprotein susceptible to ROS-induced aggregation [146].

In addition to antioxidant machinery, the physiologic tissue protective mechanisms aimed to control the hemoglobin-dependent oxidative reactions involve Hp and Hpx, two molecules which can scavenge free Hb and heme, respectively, undergoing endocytosis by macrophages. Hp covalently binds Hb dimeric subunits in blood delivering them to macrophages, through the CD163 receptors, for safe degradation. Here, the degradation of heme into carbon monoxide, bilirubin and biliverdin byproducts occurs, completing the detoxification process and preventing the Hb peroxidative secondary reactions toxic to tissues [147]. Hpx is highly concentrated in blood plasma, from 8 to 21 mM, and specifically binds the heme transporting it to the liver [148]. Importantly, the Hpx-heme complex is inactive as a pro-oxidant compound because, contrarily to the Hp-Hb complex, it is unable to bind or consume O_2_^•−^, ^•^NO and H_2_O_2_ [149]. The heme is then released through a receptor-mediated mechanism in parenchymal cells, while Hpx recycled to its intact free form and released again into the blood stream [148].

In SCD, the substantial hemolysis does not allow Hp and Hpx to complete binding with Hb and heme, respectively, becoming rapidly overwhelmed [136,150]. Both the Hp and Hpx blood concentrations were decreased in adult [71,120,123,151] as well as in pediatric SCD patients [152]. The depletion of the Hp and Hpx concentrations have been reported to favor the thrombo-inflammation in the vasculature through a mechanism involving the component C5-dependent complement activation [151], which also positively correlated with the percentage of dense sickle cells [153]. In addition, Hp and Hpx depletion also correlated with the increase of lipid peroxidation and increased concentration of oxidized LDL in the pulmonary artery [123]. With this regard, Hb/Hp, and heme/Hpx complexes have been detected in plasma lipoproteins of SCD patients, with the ApoA-1 particles of HDL being more associated with heme/Hpx [154,155] and the LDL fraction containing higher concentrations of Hb/Hpx [155]. These HDL/heme/Hpx complexes could favor a modification of the role of the lipoprotein from being anti-inflammatory to pro-inflammatory [154]. Taken together, these data demonstrate that the loss of the physiologic scavengers of Hb or heme strongly contribute to the plasma heme-mediated lipid oxidation and tissue injury in SCD.

## 8. Antioxidant Therapy for SCD

The research of suitable compounds able to (i) limit the hemoglobin-dependent oxidative reactions, (ii) scavenge reactive oxidizing species released and (iii) repair the reactive oxidizing species-mediated tissue oxidative damage is a fundamental step in the clinical management of SCD.

There are currently two types of treatment for SCD: primary treatments, which treat the root causes of the disease (gene therapy and HbF inducers antisickling agents) and secondary treatments, which target one of the downstream sequelae of HbS polymerization. In the secondary type of treatment, beside factors working against adhesion, inflammation and thrombophilia, the antioxidant therapy plays an important role for SCD treatment [156]. In fact, as already mentioned, oxidative stress can lead to disturbance of cell membranes, exposure of adhesion molecules and damage to the contents of the sickle red blood cells [157]. In Table 2 are reported the most promising antioxidant therapeutic strategies, which showed a benefit either in the reducing oxidative stress parameters or in the prevention of pathophysiologic events in SCD patients.

### 8.1. L-Glutamine

L- Glutamine is a precursor of nicotinamide adenine dinucleotide (NAD) required for antioxidant mechanism through the formation of reduced nicotinamide adenine dinucleotide (NADH) [156]. Oral administration of L-glutamine in SCD patients has been approved on July 2017 by Food and Drug Administration (FDA).

The RBC oxidative damage is most likely consequence of instability of HbS, increasing in free radical generation and impairing antioxidant defenses. This hemoglobin instability leads to denaturation of HbS, through its oxidation to methemoglobin. Methemoglobin reductases slow down this process using NADH. In sickle RBC, there is a decreased NADH/NAD ratio with a consequent decreased NAD redox potential, manifestation of a compensatory mechanism against increased oxidant sensitivity [134]. Several trials demonstrated the beneficial effects of oral administration of L-glutamine in SCD in improving cellular redox potential and facilitating protein and glutathione synthesis [157,158,159]. Finally, additional studies suggested that oral L-glutamine supplementation improves the endothelial adhesion of sickle RBC, one of the major factors involved in the pathophysiology of vessel-occlusion. The mechanism underlying this effect is still unclear; however, the improvement of NAD redox potential may protect RBC from oxidant damage and the consequent stimulation of inflammation and expression of adhesion molecules [158].

**Table 2:** Antioxidants in SCD therapy.

Antioxidants	Mechanisms	Effects in SCD Patients	Comments
L- Glutamine	Acts through the formation of reduced NADH.	Improves cellular redox potential and adhesion of sickle RBCs to the endothelium; facilitates protein and glutathione synthesis.	FDA approved; [155,156]
N-Acetylcysteine	Substrate for GSH generation.	Reduces the number of RBCs expressing phosphatidylserine, marker of peroxidative damage to inner membrane of RBCs.	NCT01800526 phase 2 trial; NCT01849016 phase 3 trial
Zinc supplementation	Zinc deficiency is associated with high incidence of infections, vaso-occlusion events and the chronic oxidative stress.	In young SCD patients improves linear growth and weight gain. Has beneficial effects on immunity, inflammatory state and oxidative stress.	NS; [159,160]
Nitric oxide	Reduced ^•^NO concentration can be associated to increased levels of free O_2_^•−^.	Inhaled ^•^NO improves tissue oxygenation and reduces pain in SCD patients with pulmonary hypertension.	NCT00094887 phase 2 trial
L-arginine	Induces GSH synthesis.	Improves ^•^NO bioavailability.	NCT02447874 phase 2 trial
Alfa-lipoic acid	Induces GSH synthesis.	Increases glutathione level.	NCT01054768 phase 2 trial
L-acetyl-L-carnitine	Improves mitochondrial metabolism, facilitating entry of long-chain fatty acids into mitochondria and decreasing lipid peroxidation in tissue.	Decreases lipid peroxidation.	NCT01054768 phase 2 trial
Gum Arabic	Acts as immuno-modulatory.	Increases total antioxidant capacity and decreased MDA and H_2_O_2_ levels.	NCT04191213 phase 2 trial
Omega-3 fatty acids	O3FA deficiency correlates with an increase in plasma levels of the inflammatory biomarker	Have beneficial effects on vascular activation, inflammation and antioxidant systems.	[156,157,158,159,160,161,162,163,164,165,166,167,168,169,170]
Curcumin	Can modulate the activity of enzymes active in the neutralization of free radicals and it can inhibit ROS-generating enzymes.	Mitigates the effects of iron induced oxidative stress on lipid peroxidation and ^•^NO levels.	[169,170]
Vitamins A, C and E	Their deficiency increases susceptibility to infection and hemolysis.	Conflicting results about the effectiveness of their supplementation on oxidative stress.	NCT03903133 phase 4 trial
Iron chelators	Avoids excessive iron overload and the consequent ROS generation.	Have a central role in the treatment of transfusion-dependent hemoglobinopathy.	NS; [158]

NADH: reduced nicotinamide adenine dinucleotide; RBCs: red blood cells; GSH: glutathione; EC: endothelial cell; ^•^NO: nitric oxide; NCT number: ClinicalTrials.gov identifier; O_2_^•−^: superoxide anion radical; MDA: malondialdehyde; ROS: reactive oxygen species; NS: not specified.

### 8.2. N-Acetylcysteine (NAC)

NAC is an important antioxidant with pleiotropic effects on inflammation and vasomotor function [156,160]. It is a substrate for the synthesis of GSH, one of the most important intracellular antioxidants and may play an important role as antioxidant treatment. Indeed, within the cytoplasm, NAC is converted to L-Cysteine, which is a precursor of GSH resulting in an increase of its concentration. GSH has been found to be 32–36% lower in RBCs from SCD patients compared to healthy controls, while some antioxidant enzymes involved in oxidant detoxification, such as SOD and Gpx, have been found significantly higher in patients with SCD [28].

In sickle cell, there is an increased consumption of GSH due to excessive reactive oxidizing species formation, resulting in a decreased ration between GSH and its oxidized form GSSG. In an open label randomized pilot study, Nur and colleagues [135] observed that NAC treatment reduced oxidative stress. In particular, they observed (i) a reduced cell membrane phosphatidylserine expression, marker of peroxidative damage to the erythrocyte inner membrane, and (ii) a decrease of AGEs and cell-free hemoglobin. The association between AGEs and the degree of hemolysis and organ complication in sickle cell patients and, on the other hand, an inverse correlation with GSH levels has been recently demonstrated. These results probably suggest that, enhancing GSH levels, NAC treatment could reduce AGEs levels and oxidative tissue damage [135].

### 8.3. Zinc Supplementation

Zinc deficiency has been implicated in SCD pathological events. Thus, this element as a therapeutic agent may be very useful in these patients. Zinc deficiency was associated with high incidence of infections connected to weakened cell mediated immunity, with vaso-occlusion events correlated with high level of endothelial cell VCAM-1 molecule and with the chronic oxidative stress. In a study of prepubertal children with SCD-SS, the therapeutic effect of zinc supplementation was evaluated on growth and body composition. Results demonstrated that young SCD patients can benefit from zinc supplementation to improve linear growth and weight gain [161]. Furthermore, in a very important double-blind, placebo-controlled study, zinc supplementation (with 25mg elemental zinc as acetate, three times a day for 3 months) ameliorated several pathophysiological parameters chronically existent in these patients: Beneficial effects were observed on immunity, inflammatory state and oxidative stress [162].

### 8.4. Nitric Oxide and L-Arginine

In addition to its role in vascular tone, blood flow and adhesion, ^•^NO is known to possess antioxidant properties. Moreover, inhalation of exogenous ^•^NO, can be used to reduce pain in SCD patients with pulmonary hypertension [86] and microvascular occlusion in different parts of the body, but controversial results are reported by randomized trials (NCT00094887) [163].

In SCD patients, ^•^NO concentration declines, and its reduced bioavailability can be associated to increased levels of free O_2_^•−^. Therapy with L-arginine has been demonstrated to improve ^•^NO bioavailability either in transgenic knockout sickle mice or in SCD patients, although in different clinical trials, conflicting results were obtained [164,165]. The cause of this result has in part been attributed to a deficiency of R-BH4 action. This is an endogenous pterin widely distributed in mammalian tissues that works as a cofactor of aromatic amino acid hydroxylases and nitric oxide synthases. In SCD patients, its deficit is implicated in the mechanism of several diseases such as atherosclerosis, hypertension, diabetic vascular disease and vascular complications [166].

### 8.5. α-Lipoic Acid and Acetyl-L-Carnitine

Other compounds with antioxidant properties are α-lipoic acid (LA) and acetyl-L-carnitine (ALCAR). LA increases glutathione level, whereas ALCAR decreases lipid peroxidation [156].

One of the mechanisms of antioxidant protection by LA is the induction of GSH synthesis, with a dose-related mechanism, through inducing Nrf2-dependent transcription of γ-glutamyl cysteine ligase (GCL), the rate-controlling enzyme in the synthesis of GSH [167,168]. On the contrary, the reason of the beneficial effect of ALCAR is not so clear; however, this might occur from improved mitochondrial metabolism, facilitating entry of long-chain fatty acids into mitochondria and decreasing lipid peroxidation in tissue. Studies suggest that this nutrient may be able to maintain the normal shape of RBCs and decrease peroxidative damage [160,168].

Finally, the evaluation of oxidative stress in human fibroblast exposed to iron excess shows an increased antioxidant effect of combination treatment with LA and ALCAR, suggesting a synergic influence of two compounds [168].

### 8.6. Other Antioxidant Agents

Gum Arabic (GA), omega-3 fatty acids and curcumin are reported to diminish oxidative stress in SCD, but their role has not been widely accepted [156].

Oral intake of GA has been shown to provide several health benefits, such as probiotic, immuno-modulatory, antioxidant and cytoprotective effects. Available experimental data show its protection against hepatic, renal and cardiac toxicities in rats and, due to its antioxidant properties, this compound may find clinical application sickle cell anemia. In a phase II trial, Kaddam and colleagues treated 47 SCD patients with 30 g/day GA for 12 weeks and demonstrated that GA significantly increased total anti-oxidant capacity and decreased MDA and H_2_O_2_-related oxidative markers [169].

Limited studies dealing with Ω-3 dietary supplementation are available. Kalish demonstrated the impact of ω-3 fatty acids on vascular activation, inflammation, and antioxidant systems. Authors assessed a modified red cell membrane composition (lower ω -6/ ω -3 ratio), a reduction of neutrophil count and beneficial effects on the cardiovascular system [170].

Curcumin could mitigate the effects of iron induced oxidative stress on lipid peroxidation and ^•^NO levels, as showed in rats exposed to iron overloaded toxicity [171,172].

Despite the observed increased susceptibility to infection and hemolysis in SCD patients with deficiency of vitamins A, C and E, conflicting results are available about the effectiveness of their supplementation on oxidative stress in these patients.

Finally, the role of iron chelators (deferiprone, deferoxamine and deferasirox) is central in the treatment of transfusion-dependent hemoglobinopathy, avoiding excessive labile iron overload and the consequent ROS generation [160]. Both in in vitro and in animal models, it has been shown that these compounds (i) decrease the RBC membrane-oxidative damage and the production of lipid oxidation product (deferiprone) and (ii) attenuate blood cell adhesion to endothelial cerebral venules (deferoxamine) [160]. Finally, in a longitudinal study, SCD patients receiving simultaneously deferasirox and hydroxyurea showed a marked decrease of plasma lipid peroxidation products as well as increased antioxidant capacity levels [173].

These promising results should encourage the development of the future research focused on the antioxidant therapy also in combination with the drug treatments. This therapeutic strategy targeted to the reactive oxidizing species-releasing pathway and limiting the intra- and extra-cellular oxidative damage, could reduce the clinical complications of the disease with particular regard to SCD-associated vasculopathy.

## 9. Conclusions

This review summarizes our present understanding on the mechanisms of oxidative stress in sickle cell disease and discusses the involvement of reactive oxidizing species in the pathophysiology of SCD and their potential implication for SCD management.

The recent discoveries in developing novel drugs have led to improved survival and decreased morbidity in patients with SCD, but an improved understanding of oxidative stress will lead to targeted therapies that should improve outcomes for this patient population.
